# Practical Departments

**Published:** 1906-01-06

**Authors:** 


### PRACTICAL DEPARTMENTS.

### MESSRS. ROBERT BOYLE AND SON'S VENTILATING APPARATUS.

Messrs. Boyle and Son, of Holborn Viaduct, have long been well known in the ventilating world in connection with their air-pump ventilator, an apparatus designed to take the place of the fan, and the heating apparatus that are sometimes employed to produce an upward draught. There are no moving parts to Messrs. Boyle's ventilator. It operates by virtue of the property, well known to ventilating engineers, by which a current of air passing across the mouth of a tube, duct, etc., exerts a sucking or exhausting action upon the fluid within the tube or vessel. It is the principle upon which the scent spray works. The ventilator, which is to be fixed on the highest ridge of the building, and connected to the room to be ventilated by a pipe, exhausts the air from the top of the room, proper, all inlets being provided with heating apparatus where required. Messrs. Boyle also construct a modification of the above, designed to provide a downward current of air, taking the place of the pressure fan. Both operate by the motion of the air in the neighbourhood, but the exhaust ventilator is protected from the possibility of down draughts by the arrangement of the cover and air-passages. The firm also make special forms of heating apparatus to be placed in the exhaust shafts in connection with their ventilators for use in washhouses, and an extension of these for destroying bacteria where required. In addition they make self-regulating air-valves, inlet and outlet ventilators of the usual form but with their special designs, radiators, and heating apparatus generally.

